# Kinetic Study on Mutagenic Chemical Degradation through Three Pot Synthesiszed Graphene@ZnO Nanocomposite

**DOI:** 10.1371/journal.pone.0135055

**Published:** 2015-08-19

**Authors:** Mohd Shoeb, Braj Raj Singh, Mohammad Mobin, Gul Afreen, Wasi Khan, Alim H. Naqvi

**Affiliations:** 1 Centre of Excellence in Materials Science (Nanomaterials), Department of Applied Physics, Z.H. College of Engg. & Tech., Aligarh Muslim University, Aligarh, Uttar Pradesh, India; 2 Department of Applied Chemistry, Z.H. College of Engg. & Tech., Aligarh Muslim University, Aligarh, Uttar Pradesh, India; 3 Department of Chemical Engineering, Indian Institute of Technology Delhi, Delhi, India; Institute for Materials Science, GERMANY

## Abstract

The study was taken up with the objective to synthesize graphene-zinc oxide nano particles (NPs) nanocomposite (Gr@ZnO-Nc) via *In-situ* synthesis method. The structural, optical, thermal, electrical and photocatalytic properties of the synthesized Gr@ZnO-Nc were studied. The characterization data confirmed that the ZnO NPs were successfully incorporated into the graphene sheets. Further, TGA/DTA results exhibited an enhanced thermal stability of the Gr@ZnO-Nc compared with the graphene. The Gr@ZnO-Nc, graphene sheets were uniformly wrapped by ZnO NPs, which can protect graphene and delay their oxidation in air. The synthesized Gr@ZnO-Nc was used for the efficient photodegradation of a carcinogenic methyl orange (MO) dye. The results exhibited promising photodegradation of the MO dye under UV light irradiation through the production of reactive oxygen species (ROS). The promising effect of Gr@ZnO-Nc on the photodegradation properties was conferred by the large surface area which increased adsorption capacity, and the strong electron transfer ability. Thus, it is encouraging to conclude that the synthesized Gr@ZnO-Nc has environmental significance with its utility in remediation in the hazardous MO dye.

## Introduction

Graphene, a single atom thin carbon sheet with a hexagonal packed lattice structure, which has attracted huge research interest in both basic and applied fields of science[1]. The graphene and metal oxide based nanocomposites materials are a class of useful materials with unique physical, chemical and electronic properties, which are closely related to their size, shape, elemental composition and structure[1, 2]. Immense progress has been made in the synthesis of novel graphene and metal oxide based nanocomposites materials and their potential applications in diverse fields, importantly in photocatalysis[2, 3]. The graphene and metal oxide based nanocomposites materials offer a powerful way to enhance the application of graphene by enabling versatile and tailor-made properties with high performance far beyond those of the graphene [3, 4]. In literature, the improved photo catalytic properties of graphene-metal oxide NPs nanocomposite materials have been reported, which has been attributed to the good electron conduction of graphene [5–9]. Graphene and metal oxide based nanocomposites materials can significantly enhance their photocatalytic properties[10]. Therefore, Graphene and metal oxide based hybrid nanostructured materials enhanced photocatalytic properties and these properties have been extensively explored recently for the degradation hazardous dyes [3].

It is well-known that zinc oxide nanoparticles (ZnO NPs) have numerous potential applications like optoelectronic[11–13], environment[14–18], biomedicine[14, 19–21] due to its wide energy bandgap (3.37 eV) and large exciton binding energy (60 meV)[22]. In recent years, large amount work have on the development of efficient photocatalysts using heterojunction of ZnO NPs with graphene, because graphene possesses unique physical properties as well as larger surface areas[3]. In addition, the production cost of graphene sheets in large quantities is much lower than that of other nano carbon allotropes (CNTs and fullerenes)[23, 24]. Therefore, graphene as a low cost alternative to CNTs and fullerenes in nanocomposites materials synthesis and are being used commonly for environmental application [22, 25, 26].It is expected that graphene composite with ZnO NPs will improve the charge transfer between the metal oxides and methyl orange (MO) dye molecules. The intrinsic properties of the graphene have been modified through the synthesis of their hybrid nanocomposites with the addition of ZnO NPs [27] [28–30]. Therefore, it is of great significance to obtain a graphene-ZnO NPs nanocomposite possessing efficient photocatalytic activity well beyond pure ZnO NPs, which could be potent in environment remediation. Therefore, in this study we demonstrated a facile and reproducible route to obtain a chemically bonded Graphene-ZnO NPs nanocomposite (denoted as Gr@ZnO-Nc) *via* a three step method. The synthesized Gr@ZnO-Nc was investigated for their photocatalytic activity against the carcinogenic MO dye under UV light irradiation.

## Material and Methods

### 2.1 Materials

Commercially available natural graphite powder (Sigma Aldrich, USA) was used as a source material for graphene oxide synthesis. In addition, 37% hydrochloric acid (HCl), 98% sulphuric acid (H_2_SO_4_), 30% hydrogen peroxide (H_2_O_2_), potassium permanganate (KMnO_4_), sodium nitrate (NaNO_3_), zinc acetate dihydrate [Zn(AcO)_2_.2H_2_O], sodium hydroxide (NaOH), sodium borohydride (NaBH_4_) and methyl orange (C_14_H_14_N_3_NaO_3_S) chemicals were obtained from the local suppliers. All reactants were used without further purification.

### 2.2 Gr@ZnO-Nc Synthesis

Graphene oxide (GO) was synthesized from 2 g of natural graphite powder using a modified Hummer’s method[31]. The synthesized GO was centrifuged and washed successively with 4% HCl until to attained neutral pH value. The synthesized GO was dried in vacuum oven at 70°C for overnight and GO synthesis was confirmed through the absorbance, XRD and FTIR analysis. The obtained GO was used for the synthesis of Gr@ZnO-Nc following the slightly method described by Li and Huaqiang Cao [10]. In the first step of synthesis, ~1.0 g of GO powder was added into 100 mL water and ultrasonicated for 10 min at room temperature. To the obtained solution, 1 mM aqueous solution of Zn (AcO)_2_. 2H_2_O was added and stirred for 30 min at 50°C for the complete exchange of ions. In the second step of synthesis, NaOH was added for facilitating the conversion of Zn salts to ZnO NPs. Thus, the alkaline pH of the synthesis reaction mixture leads to the rapid removal of functional groups in GO, which have been reported to be unfavorable for the effective combination of ZnO NPs with graphene [32]. Consequently, the ZnO NPs was present in the soluble form rather than on the partially reduced graphene. In the third step of synthesis, additional reducing agent (NaBH_4_) was added to restore GO to fully reduced graphene. Both of them resulted in a redundant process and the poor dispersion of ZnO NPs on graphene. The final synthesized reaction mixture was stirred for 1h at two 150°C and synthesized Gr@ZnO-Nc was collected through centrifugation. The synthesized Gr@ZnO-Nc was washed several times with water and ethanol thoroughly and finally dried and stored in amber colour sample container until further use [33].

### 2.3. Gr@ZnO-Nc Characterization

The optical characteristic of the Gr@ZnO-Nc was measured in the ethanol solution by measuring the absorbance (A) using UV-vis spectrophotometer (Perkin Elmer Life and Analytical Sciences, CT, USA) in the wavelength range of A_200 to 800_ nm [34]. The vacuum dried Gr@ZnO-Nc powder was stored in amber color vials at room temperature under dry and dark conditions until used for further characterization. The X-ray diffraction (XRD) patterns of powder sample of Gr@ZnO-Nc was recorded on MiniFlex TM II benchtop XRD system (Rigaku Corporation, Tokyo, Japan) operating at 40 kV and a current of 30 mA with Cu Kα radiation (λ = 1.54 A^0^). The diffracted intensities were recorded from 5° to 80° 2θ angles [33]. The crystalline size (D) of the ZnO-NPs in Gr@ZnO-Nc was calculated following the Debye-Scherrer formula:

$$D=\frac{0.9\lambda }{\beta COS\theta }$$

Where λ is the wavelength of X-ray, β is the broadening of the diffraction line measured half of its maximum intensity in radians and θ is the Bragg’s diffraction angle. The crystalline size of the ZnO NPs was determined by employing the full width at half maximum (FWHM) value of the (101) XRD peak present in the Gr@ZnO-Nc. The scanning electron microscopy (SEM) was carried out using the fine powder of the Gr@ZnO-Nc on a carbon tape in a JSM 6510LV scanning electron microscope (JEOL, Tokyo, Japan) at an accelerating voltage of 20 kV. The elemental analysis of Gr@ZnO-Nc was determined using the Oxford Instruments INCAx-sight energy dispersive X-ray (EDAX) spectrometer equipped SEM [35]. The transmission electron microscopy (TEM) of Gr@ZnO-Nc was carried out on JEOL 100/120 kV TEM (JEOL, Tokyo, Japan) with an accelerating voltage of 200 kV [36]. For the atomic force microscopy, thin film of the Gr@ZnO-Nc was prepared on the borosilicate glass slide to see the surface morphology. The prepared thin film was analyzed on the atomic force microscope (AFM; Innova SPM, Veeco) in tapping mode. The commercial etched silicon tips as scanning probes with typical resonance frequency of 300 Hz (RTESP, Veeco) was used. The microscope was placed on a pneumatic anti-vibration desk, under a damping cover and analysis was performed using the SPM Lab software [37, 38]. The images of electron microscopies of EDAX and AFM were obtained and converted into an enhanced meta file format. For the functional characterization of the Gr@ZnO-Nc, powder was mixed with spectroscopic grade potassium bromide (KBr) in the ratio of 1:100 and spectra recorded in the range of 400–4000 wavenumber (cm^-1^) on Perkin Elmer FT-IR spectrometer Spectrum Two (Perkin Elmer Life and Analytical Sciences, CT, USA) in the diffuse reflectance mode at a resolution of 4 cm^-1^ in KBr pellets [39]. The functionalization and thermal stability of the Gr@ZnO-Nc was investigated by thermogravimetric analysis (TGA) (Perkin Elmer Pyris 1 TGA Thermogravimetric Analyzer) at a heating rate of 10°C/min under nitrogen atmosphere [33]. Frequency dependent impedence spectroscopy measurements were performed in the frequency range of 75kHz to 7MHz using a LCR meter (Agilent-4285A). The pellets were coated on adjacent faces with silver paste, thereby forming parallel plate capacitorgeometry [40].

### 2.4. Gr@ZnO-Nc photocatalytic activity measurement

The photocatalytic activity of Gr@ZnO-Nc was measured against MO dye under UV light irradiation [38]. In the photocatalytic photodegradation experiment, 20 μg/mL of Gr@ZnO-Nc catalyst was added to 100 mL dye solution (25 μgmL^−1^). Before irradiation, the suspensions containing MO dye and Gr@ZnO-Nc were magnetically stirred in the dark for 60 min to ensure the establishment of an adsorption/desorption equilibrium. At a fixed time interval (up to 40 min) 5 mL aliquots were sampled and then magnetically separated to remove essentially all the Gr@ZnO-Nc. The filtrate was analyzed by recording variations in the maximum absorption band (A_465_ nm) using a UV-vis spectrophotometer (Perkin Elmer Life and Analytical Sciences, CT, USA) in the wavelength range of A_200 to 800_ nm. The active species generated in the photocatalytic system were measured by trapping with *tert*-butyl alcohol (C_4_H_10_O) and disodium ethylenediaminetetraacetate dehydrate (EDTA-Na_2_;C_10_H_14_N_2_Na_2_O_8_·2H_2_O). The photodegradation of the MO dye via the photocatalytic activity of Gr@ZnO-Nc was calculated following the formula:

$$\text{Photodegradation efficiency}\;\left(\%\right)=\frac{Co\;-C}{Co}X100$$

Where the Co is the initial concentration of MO dye before photodegradation and C is the absorbance after the different time intervals.

## Results and Discussion

### 3.1. Gr@ZnO-NC Synthesis

**Fig 1:**
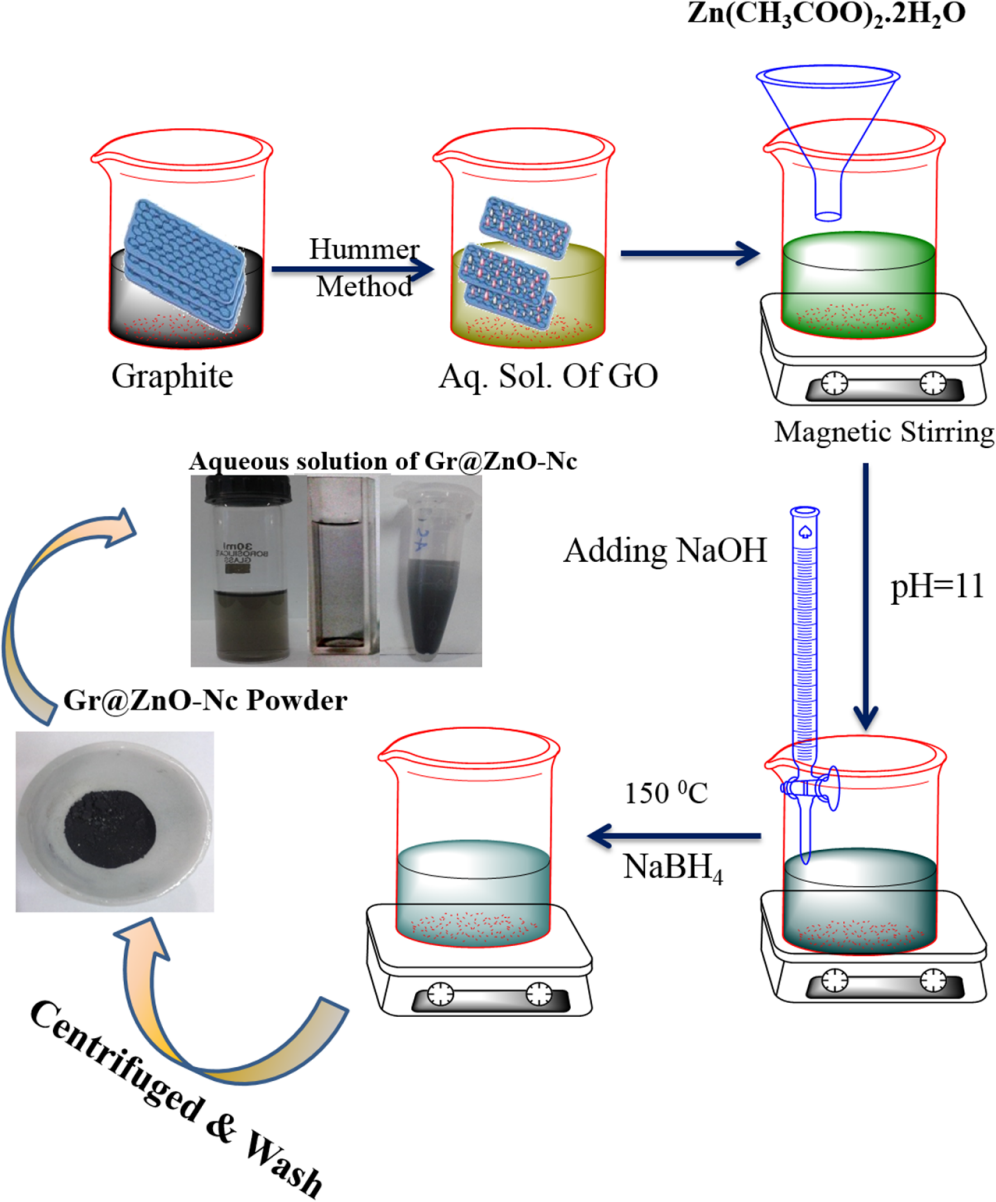
Gr@ZnO-Nc *in-situ* synthesis using three step method.

The GO was synthesized employing the Hummer's method and collected by centrifugation. Under specific conditions, the GO to Gr@ZnO-NC was synthesized by the sodium borohydride reduction method. A Gr@ZnO-NC was prepared using Zn (AcO)_2_.2H_2_O adsorbed GO sheets as precursors. In this Gr@ZnO-NC, in situ formed ZnO NPs were derived from the adsorbed Zn (AcO)_2_. 2H_2_O which were attached to graphene sheets to prevent their aggregation. The as-synthesized Gr@ZnO-NC showed that the ZnO NPs distributed randomly on the graphene sheets due to the template effect of GO. The synthesis Gr@ZnO-NC was indicated by the gradual colour change of the mixture as the initial dark yellow solution quickly turned dark brown and eventually became black within 20 min. The colour change and formation of black insoluble particulate material suggested the successful synthesis of Gr@ZnO-NC (Fig 1).

**Fig 2:**
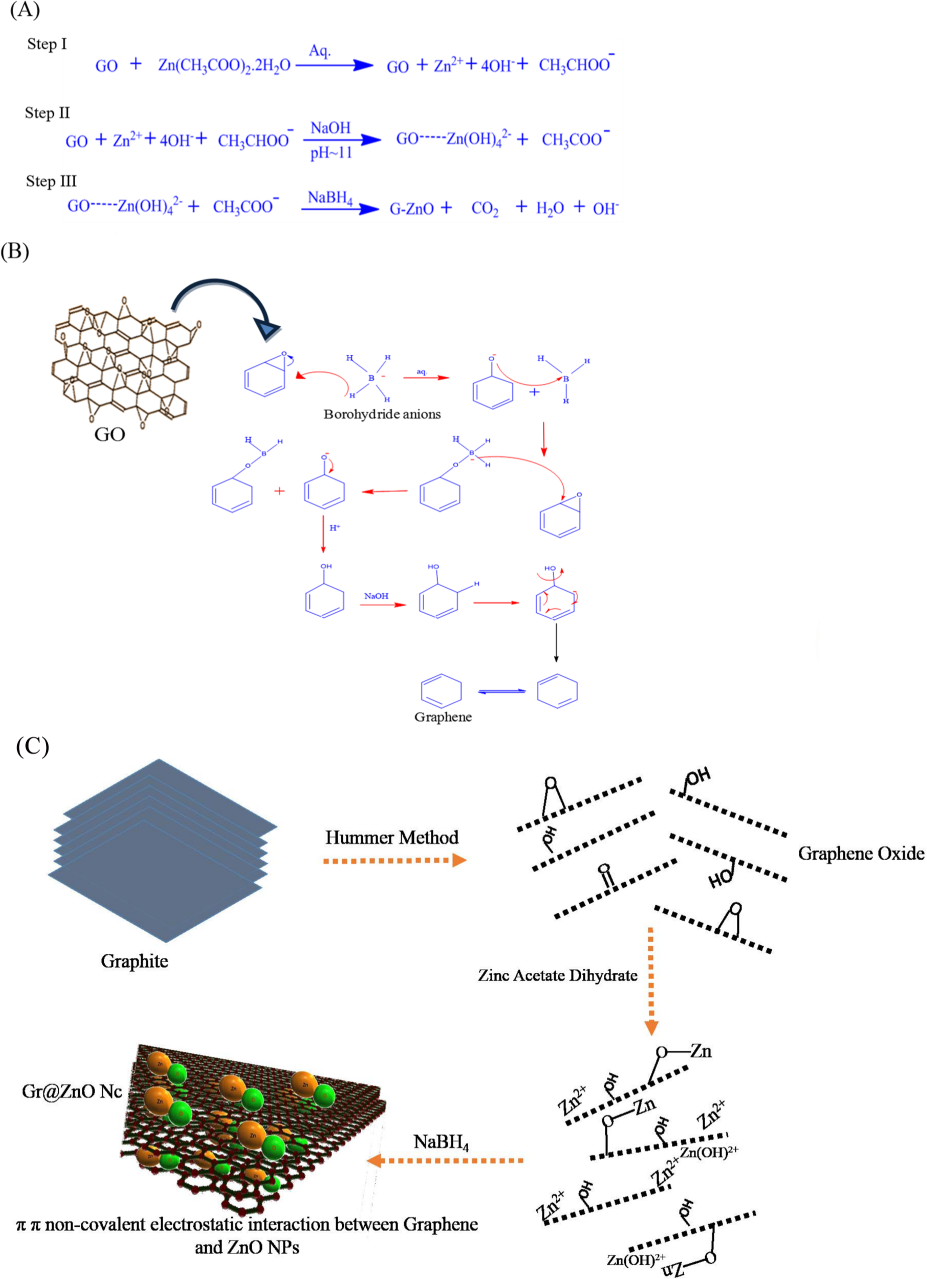
(A) Possible chemical reactions of Gr@ZnO-Nc *in-situ* synthesis using three step method. (B) Role of NaBH4 in the reduction of GO to Graphene *in-situ* synthesis of Gr@ZnO-Nc. (C) Schematic for the preparation of Gr@ZnO-Nc and interaction between Graphene and ZnO NPs.

**Fig 3:**
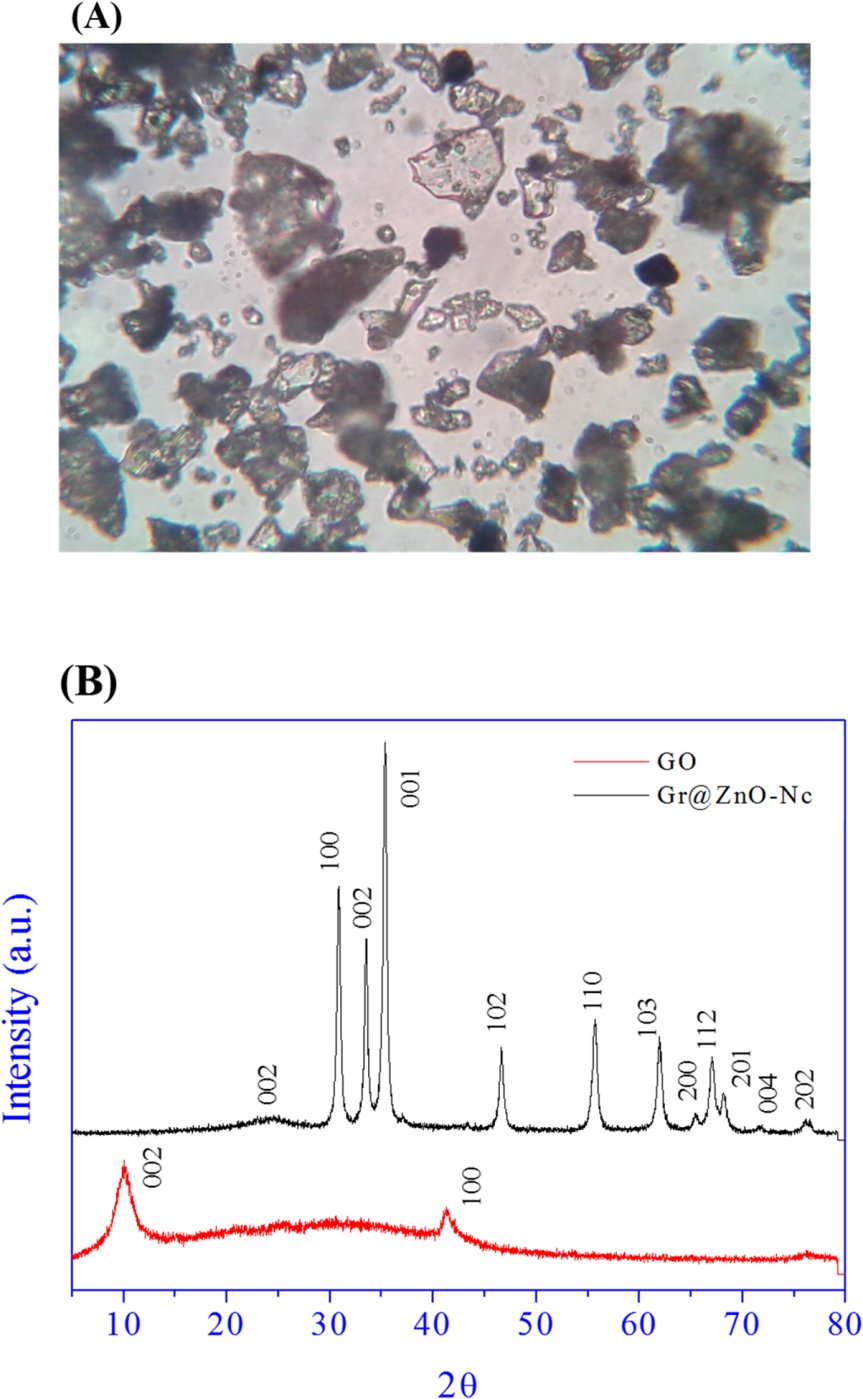
(A) Gr@ZnO-Nc optical microscopic image at 40X magnification. (B) XRD spectrum of synthesized GO and Gr@ZnO-Nc.

According to the plausible mechanism of Gr@ZnO-Nc synthesis is shown in Fig 2A, nucleation begin hydrothermally when Zn^+2^and OH^-^ reach the critical value of the super-saturation according to the classical theory of nucleation, which form Zn(OH)_2_ into the synthesis reaction [41]. However, the size and morphology of the ZnO NPs were affected from one region to another depending upon the nature and concentration of ions incorporated between graphene. Further, the Zn(OH)_2_ was converted into ZnO NPs by the addition of NaOH as it not only increased the concentration of OH^-^ ions in the solution to accelerate the formation of smaller size ZnO NPs, but also the higher concentration of OH^-^ ions engraved the larger size ZnO NPs from the partially reduced graphene. However, the smaller size ZnO NPs means less area of (100) surface, resulting in less energy difference between the top and side surfaces, thus they could not be engraved [41, 42]. Sodium borohydride (NaBH_4_), which is a salt containing a tetrahedral BH_4_^−^ anion and one of the most common reducing agent was added to restore GO to fully reduced graphene and detailed synthesis reaction mediated by NaBH_4_ is shown in Fig 2B. In the presence of an electrophile such as carbonyl functionality, the BH_4_^−^ anion readily performs a hydride transfer reaction resulting in an oxyanion and an electron-deficient BH_3_ molecule. Subsequent stabilization of the BH_3_ molecule with the oxyanion reinstates the BH_4_^−^ anion as a hydride transfer agent (Fig 2C). This is ideally the case until all the B–H bonds are exhausted [43]. Both of them result in a redundant process and the poor dispersion of ZnO NPs on graphene [44]. The π electrons of the carbon atom in the graphene to form the delocalized π bonds, and some unpaired π electrons could bond with the free electrons on the surface Zn^2+^ to form a Zn-O-C structure. The affinity of graphene to Zn^2+^ through an electrostatic force, thus providing more active growth sites for ZnO NPs [3, 45].Furthermore, these factors could play an important role in the generation of electronic species (e^-^ and h^+^), which may be attributed to the higher photocatalysis activity of the Gr@ZnO-Nc. The Gr@ZnO-NC synthesis was simultaneously monitored by using optical microscope. The optical microscope image depicted the transparent graphene nanosheets held the agglomerated ZnO NPs (Fig 3A).

### 3.2. Gr@ZnO-NC structural characteristics

X-ray diffraction (XRD) measurements were employed to investigate the phase and structure of the synthesized graphene oxide (GO) and Gr@ZnO-Nc. The XRD pattern of GO and Gr@ZnO-Nc are showed in Fig 3B. In the GO sample one predominant diffraction peak was observed at 10.4° which corresponds to (002) reflection plane of GO with basal spacing of d_002_ = 8.26 Å [46, 47]. The basal spacing of GO was higher than the graphite (3.35 Å) due to intercalation of functional groups (C-OH, C-O, C-OOH) which increased the interlayer distance. The XRD pattern of the Gr@ZnO-Nc is shown in Fig 3B; there were eleven main diffraction peaks located at 2θ = 31.34°, 34.01°, 35.44°, 46.63°, 55.78°, 61.97°, 65.56°, 67.18°, 68.24°, 71.67°, and 76.26° which correspond to the crystal planes [100] [2], [101], [102], [110], [103], [200], [112], [201], [4] and [202] of the hexagonal wurtzite structure of ZnO reported in JCDDS card (No. 36–1451, a = 3.249 Å, c = 5.206 Å), respectively [48, 49]. The XRD data of Gr@ZnO-Nc indicated that the absence of the any impurities, which attested its high quality. The average crystallite size (*D*) of ZnO NPs was calculated following the Debye-Scherrer formula:

$$D=\frac{0.9\lambda }{\beta COS\theta }$$

Where *k* = 0.9 is the shape factor, λ is the X-ray wavelength of Cu Kα radiation (1.54 Å), θ is the Bragg diffraction angle, and β is the full width at half maximum height (FWHM) of the (101) plane diffraction peak. The calculated average particle size was found to be ~20 nm of ZnO NPs in the synthesized Gr@ZnO-Nc. Further in the main characteristic diffraction peak was observed in the synthesized Gr@ZnO-Nc at 24.53° which corresponds to (002) reflection plane of graphene with basal spacing of d_002_ = 3.62 Å [47, 50]. The XRD data clearly indicates the successful synthesis of Gr@ZnO-Nc in this study.

**Fig 4:**
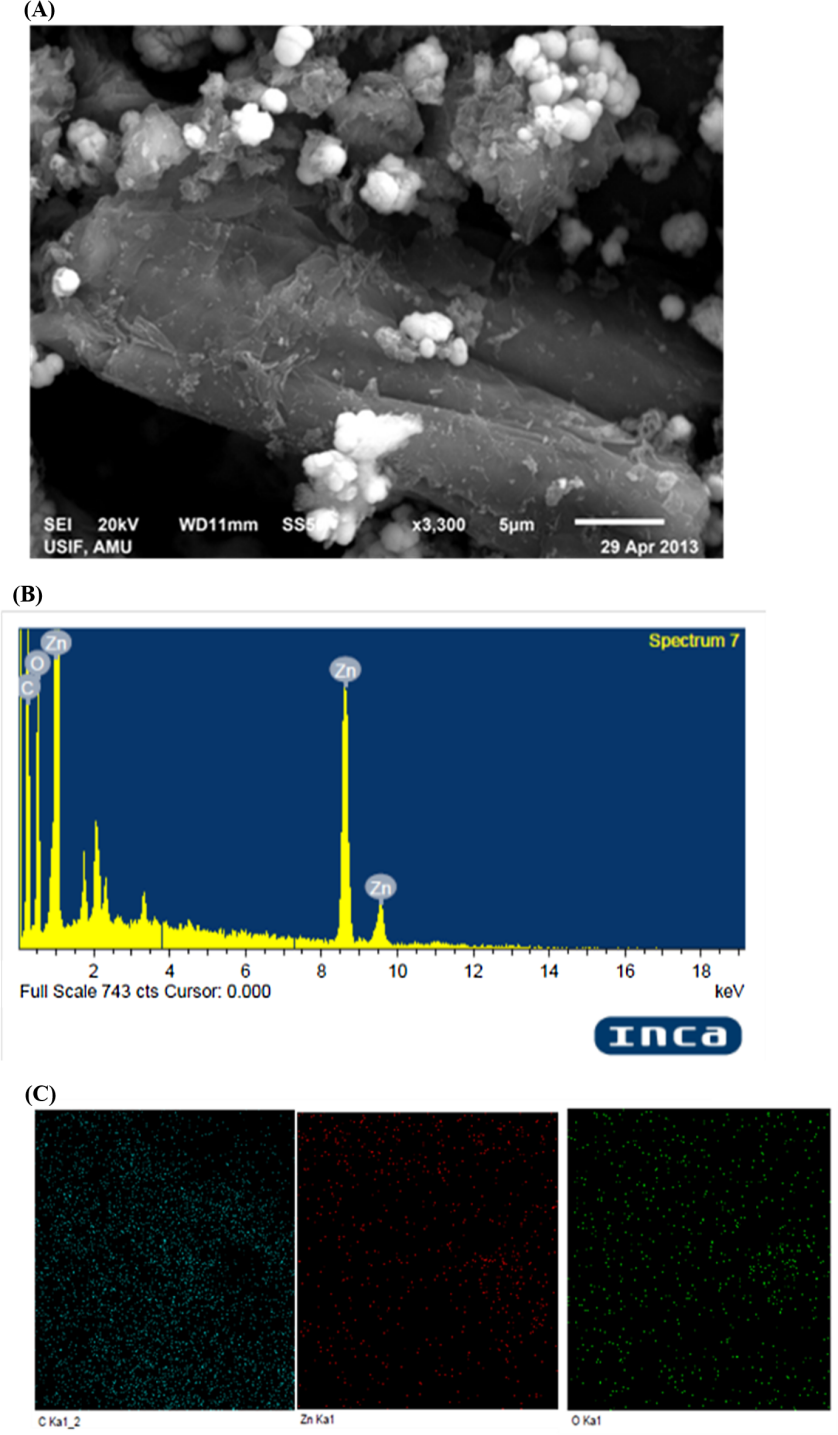
(A) Gr@ZnO-Nc SEM micrograph at 5 μm scale. (B) Gr@ZnO-Nc EDAX spectrum and elemental mappings.

**Fig 5:**
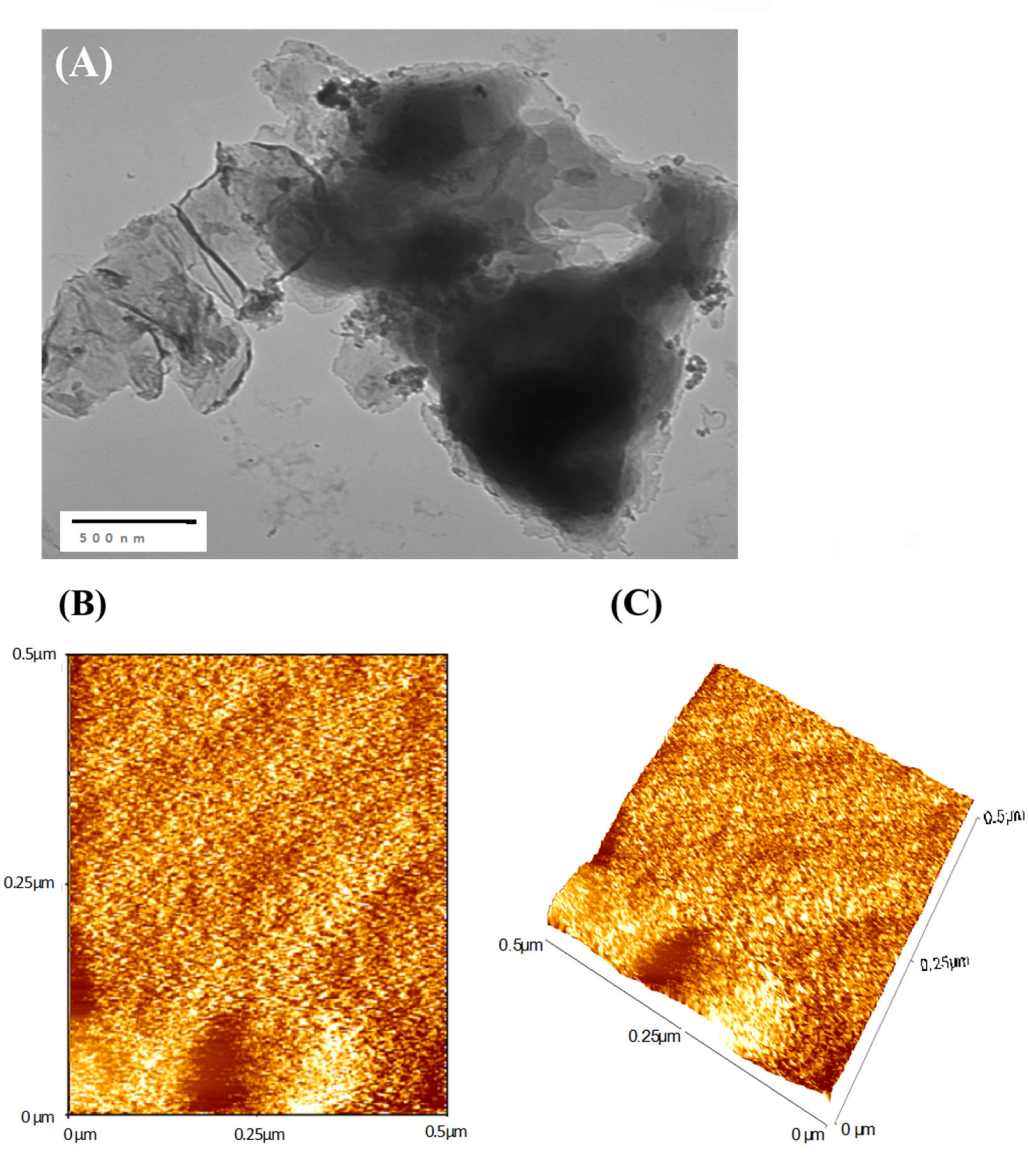
(A) Gr@ZnO-Nc TEM micrograph at 500 nm scales. (B) Gr@ZnO-Nc AFM 2D micrograph. (C) Gr@ZnO-Nc AFM 3D micrograph.

**Fig 6:**
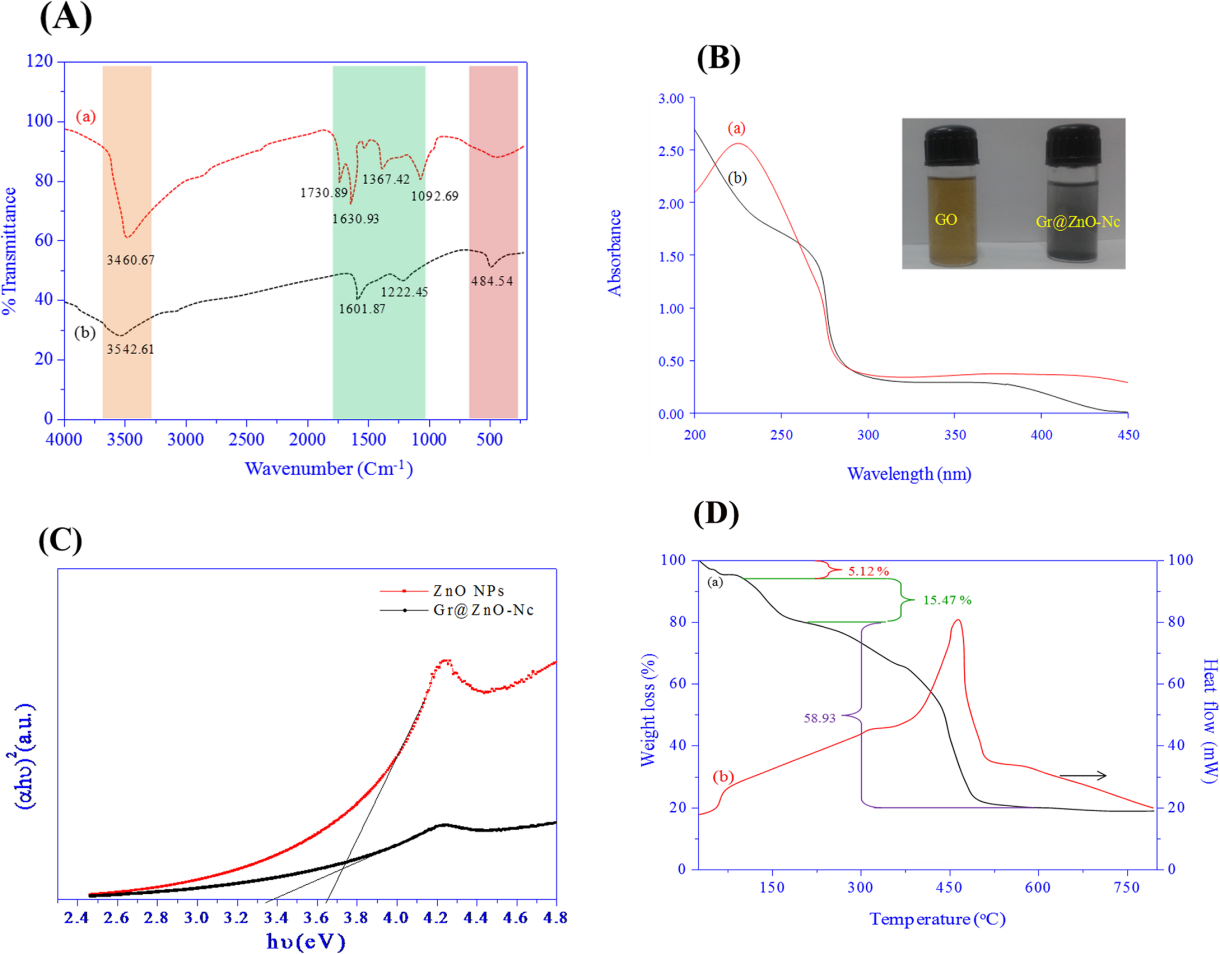
(A) FTIR spectrum of GO and Gr@ZnO-Nc. (B) UV-Visible absorbance spectra of of GO and Gr@ZnO-Nc. (C) Tauc plot depicted the energy band gap (Eg) of ZnO NPs and Gr@ZnO-Nc. (D) TGA and DTA spectra of Gr@ZnO-Nc.

Under optimized conditions, well-dispersed ZnO with a small size could be grow on the GO-derived graphene. The structure of Gr@ZnO-NC was studied by SEM, TEM and AFM analysis. As seen from the SEM image ZnO NPs are distributed uniformly on graphene sheets (Fig 4A).The corresponding energy-dispersive X-ray spectrum (EDAX) of Gr@ZnO-Nc sample showed that the presence of Zn, C, and O elements, which further proved the absence of any impurities in the sample. Results of SEM examination combined with the corresponding EDAX mappings for the elements Zn and O are shown in (Fig 4B & 4C), which further confirmed the uniform distribution of Zn and C throughout the sample. The TEM microphotographs revealed the presence of abundant irregular graphene sheets. ZnO NPs in the range of ~20 nm (Fig 5A) randomly anchored and agglomerated onto the surfaces of the graphene sheets. The AFM images also shows the irregular spherical surface morphology of Gr@ZnO-Nc (Fig 5B and 5C), which is consistent with results obtained by the XRD, SEM and TEM. The irregular spherical surface morphology observed may be due to the impregnation of the ZnO NPs in to the graphene sheet. Fig 6A shows the FTIR spectra of GO (a) Gr@ZnO-Nc (b) samples. The GO characteristic absorption bands of oxide groups at 3460.67, 1730.89, 1630.93, 1367.42, and 1092.69 cm^-1^ were assigned to the-OH stretching vibrations, C = O stretching vibrations in carboxylic acid, skeletal vibrations of unoxidized graphitic domains, O-H deformations of the C-OH groups, and C–O (alkoxy) stretching vibrations, respectively [47, 51]. Compared to the GO, the spectrum of Gr@ZnO-Nc showed the absence of the characteristic peaks of carboxyl group at 1730.89 cm^-1^, hydroxide group at 1367.42 cm^-1^ and C–O (alkoxy) groups at 1092.69 cm^-1^ while some of them decreased dramatically, indicating that most oxygen-containing functional groups in the GO were removed [52, 53]. The spectrum of Gr@ZnO-Nc showed an absorption band at 1601.87 cm^−1^, C = C stretching, indicating the restoration of the graphene network on reduction. The presence of the absorption band at 484.54 cm^-1^ of Gr@ZnO-Nc is identified to the vibration of Zn-O belonging to ZnO NPs [54, 55].

### 3.3. Gr@ZnO-NC optical characteristics

The UV visible spectra of GO (a) Gr@ZnO-Nc (b) are shown in Fig 6B. The GO sample showed the absorption peak at ~221 nm and a shoulder at ~280 nm. The peak at 221 nm is assigned to the pi to anti-pi (π → π*) transition of the aromatic C–C bonds and the shoulder at 280 nm to the non bonding to anti-pi (n → π*) transitions of the C = O bonds [56–58]. The UV spectrum of Gr@ZnO-Nc showed two distinct peaks at ~270 and ~370 nm which corresponds to the excitation of the π- plasmon of the graphitic structure and ZnO NPs characteristics, respectively. The graphene absorption peak was entirely different from GO peak and red shift to 270 nm indicated fully reduced graphene from GO [47, 59–61]. The absorption data again confirmed the successful synthesis of Gr@ZnO-Nc in this study.The UV–vis spectra reveal a characteristic absorption peak for ZnO NPs at a wavelength of ~378 nm (S1 Fig), which can be assigned to the intrinsic band-gap absorption of ZnO NPs, owing to the electron transitions from the valence band (VB) and conduction band (CB) (2_p_—Zn_3d_). The absorption peak position of the UV–vis spectrum of the ZnO NPs has been affected by the GO may be due to the absorption contribution from reduced graphene, the increase in the surface charge of the ZnO NPs, and the change of the basic process of electron–hole pair formation during GO reduction [62].

The electronic band gap (E_g_) of the ZnO NPs and Gr@ZnO-NC were determined by employing Tauc relationship as given below.

$$\alpha h\vartheta =A{\left(h\vartheta -E_{g}\right)}^{n}$$

Where ‘α’ is the absorption coefficient (α = 2.303A/t, here ‘A’ is the absorbance and ‘t’ is the thickness of the cuvett), B is a constant, ‘h’ is Planck’s constant, ‘ν’ is the photon frequency, and ‘E_g_’ is the optical band gap. The value of n = 1/2, 3/2, 2 or 3 depending on the nature of the electronic transition responsible for absorption and n = 1/2 for direct band gap semiconductor. An extrapolation of the linear region of a plot of (αhν)^2^ on the Y-axis versus photon energy (hν) on the X-axis gives the value of the E_g_ as shown in Fig 6C.The E_g_ of the Gr@ZnO-NC was found to be 3.36± 0.1 eV, compared to 3.65± 0.1 eV for bare ZnO NPs. Decrease of band gap of bare ZnO NPs might be due to the introduction of graphene which is full of zero band gap (theoretically calculated) thereby producing metastable stages VB and CB [63].

### 3.4. Gr@ZnO-NC thermal characteristics

The TGA/DTA curves of Gr@ZnO-Nc are shown in Fig 6D; the weight loss of 5.12% occurring at about 65°C is associated to adsorbed water [49]. Pyrolysis of the labile oxygen-containing functional groups at about 200°C accounts for 15.47% of weight loss. The thermal decomposition observed in the temperature range 200–500°C with 58.93% of weight loss can be verified by the DTA curve, which shows an exothermic peak at 480°C, attributed to the pyrolysis of the carbon skeleton [50, 64, 65]. These results illustrate that Gr@ZnO-Nc has a remarkable thermal stability as compared to the GO.

### 3.5. Gr@ZnO-Nc photocatalytic activity measurement

**Fig 7:**
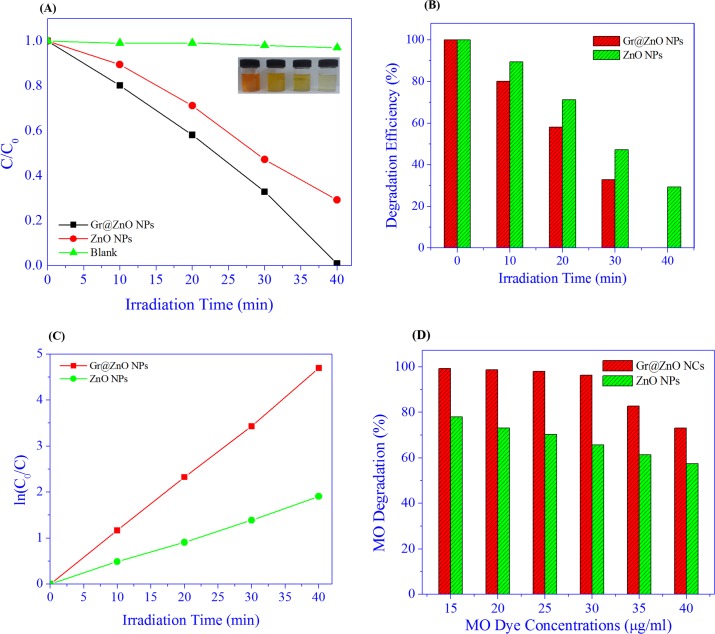
(A) Photocatalytic degradation of MO Dye under the irradiation of UV light over Gr@ZnO-Nc, ZnO NPs and without any catalysts. (B) The graphs depicting the time dependent MO Dye photo-degradation efficiency of the Gr@ZnO-Nc and ZnO NPs. (C) Plot of ln(C_0_/C) as a function of UV irradiation time for photocatalysis of MO Dye containing Gr@ZnO-Nc and ZnO NPs. D) Concentration dependent photo-degradation efficiency result of MO dye solutions in the presence of Gr@ZnO-Nc (20 μg/mL) after 40 min irradiation.

The MO dye is nowadays a serious concern for the environmentalists because of causing serious environmental pollution and affecting the public health due to their resistance to biodegradability and transformation into genotoxic and carcinogenic amines[66]. In this study we have tested the photocatalytic activity of the synthesized Gr@ZnO-Nc and ZnO NPs using MO dye as a model dye. To finish the test of the photocatalytic reaction, the following three steps were involved. In first step, the adsorption of samples was stirred in the dark for 30 min, in which the adsorption of MO dye on the Gr@ZnO NCs and ZnO NPs reached equilibrium. This helped in eliminating the effects of the adsorption of Gr@ZnO NCs and ZnO NPs for MO dye at following UV light irradiation process. In the second step, the photocatalysis of the MO dye was assessed using the Gr@ZnO-Nc and ZnO NPs upon the exposure of UV light irradiation. The photocatalytic experiments were conducted after the proper adsorption of the MO dye on the Gr@ZnO-Nc and ZnO NPs. To test the MO absorption on Gr@ZnO NCs and ZnO NPs, experiments were carried out under the same conditions to test the photodegradability of MO dye by the Gr@ZnO NCs and ZnO NPs catalysts and the results are presented in Fig 7A. Dye is resistant to self photolysis and for the same experiment with samples in the dark, a small decrease in dye concentration was observed due to the adsorption of dye on the catalyst. MO dye underwent almost complete degradation (about ~99.05%) in the presence of Gr@ZnO NCs, UV light in 40 min whereas the ZnO NPs showed the 70.77% degradation, in 40 min. This shows that Gr@ZnO NCs is more efficient in MO dye degradation than ZnO NPs photocatalysts. Color changes at different irradiation times shown in vials indicate the dye degradation (Fig 7B). The UV spectral changes of MO dye at different irradiation times with Gr@ZnO NCs and ZnO NPs catalyst (data not shown). There was a gradual decrease in intensity without the appearance of new absorption peaks. This revealed that the intermediates formed during degradation did not absorb at analytical wavelength. Photocatalytic activity was influenced by many factors in which specific surface area and the transport properties of photoinduced charge carriers were two key factors. The Gr@ZnO NCs was fairly active for photocatalytic degradation on MO dye, because it had small particle size, larger surface area to facilitate more efficient contact of the graphene sheet with mutegenic-disrupting chemical MO dye and thus resulting in enhanced photocatalytic activity. The comparative analysis of Gr@ZnO NCs with reported data graphene Graphene/ZnO nanocomposites materials is presented in S1 Table. The photocatalysis rates fit a first-order model well, that is, the integral equation of ln(C_0_/C_t_) = k_obst_ describes the tendency well, where C_0_ and C_t_ are the concentration of MO dye at time 0 and t, respectively. The k_obs_ was the observed pseudo first-order rate constant and t is the reaction time. The kinetics of degradation of MO dye under UV irradiation was also investigated. Fig 7C shows a relationship between ln(C_0_/C) and reaction time. From the results it could be said that Gr@ZnO NCs and ZnO NPs showed linear lines which indicated the photocatalytic follows pseudo first order kinetics. The apparent rate constants were determined as 11.637×10^−2^ and 4.696×10^−2^ min^-1^ for Gr@ZnO NCs and ZnO NPs, respectively. The photocatalytic activity of the Gr@ZnO NCs was about 2.47 times higher than ZnO NPs. The Kinetics of the photocatalysis rates of MO dye concentrations was also calculated, to make further mathematic inferences clear. All the relating kinetic parameters for MO dye at different concentrations are shown in Fig 7D and Table 1. The data clearly showed that the rate constant was inversely proportional to the initial concentration of MO dye. The decrease of photocatalysis efficiency and k_obs_ and as increasing the initial MO dye concentration shows that there is a competition between the instantaneous intermediates and MO dye for Gr@ZnO NCs. The promising photocatalysis rates of MO dye observed in this study may be attributed due to the high surface area of the Gr@ZnO NCs, which leads to the enhancement of the rapid separation efficiency of photo-induced electrons (e^-^) and holes (h^+^)through the electronic interaction between ZnO NPs and graphene [38].

**Table 1:** The kinetic constants of MO dye photodegradation by the Gr@ZnO-Nc and ZnO NPs at 40 min.

S.No	C_0_ (g mL^-1^)	Kinetic constant (k) (Min^-1^)	
		Gr@ZnO NCs	ZnO NPs
1	15	0.12369	0.03785
2	20	0.10896	0.03283
3	25	0.0978	0.0303
4	30	0.08242	0.02668
5	35	0.04382	0.02373
6	40	0.03284	0.02138

### 3.6. Efficient charge separation and Transportation

**Fig 8:**
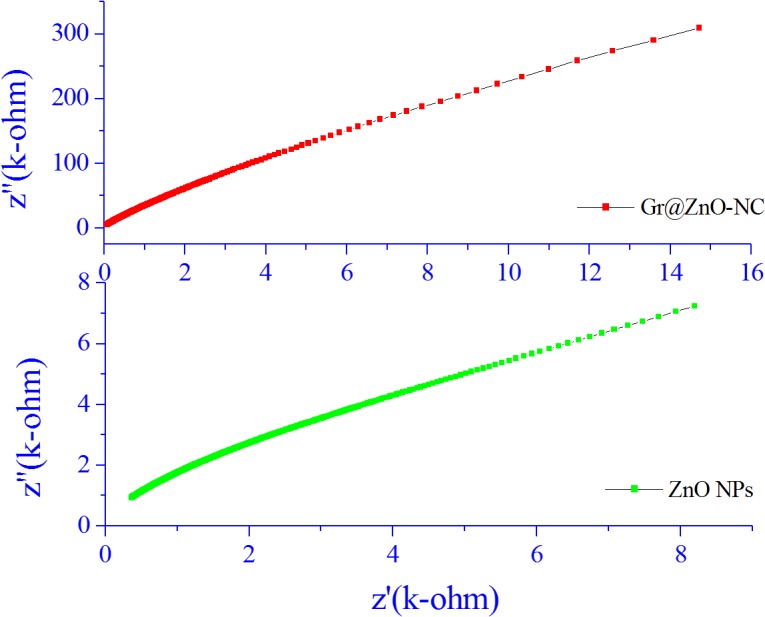
Nyquist plot of ZnO NPs and Gr@ZnO-Nc.

The graphene has outstanding conductivity due to its two-dimensional planar structure[67]. Therefore, the rapid hopping of charge carriers could be achieved and an effective charge separation subsequently accomplished. As shown in Fig 8, the electrochemical impedance spectra were presented as Nyquist plots, and it is observed that, with the incorporation of graphene, though in small amount, the semicircle in the plot became shorter, which indicated a decrease in the solid state interface layer resistance and the charge transfer resistance on the surface[68]. Overall, both the electron-accepting and hopping properties of graphene in the Gr@ZnO-Nc could contribute to the suppression of charge recombination, and thereby a higher rate in the photocatalysis would be achieved.

The electrical behaviour of ZnO NPs and Gr@ZnO-Nc was studied over a wide range of frequencies at room temperature using impedance spectroscopy. When the impedance data of materials having capacitive and resistive components was plotted in a complex plane plot it appeared in the form of a sequence of semicircles representing electrical phenomenon due to bulk (grain) material, grain boundary, and interfacial phenomenon if any. Generally, the grains are effective in high frequency region while the grain boundaries are effective in low frequency region [40].The impedance spectrum is usually represented as imaginary component of impedance (Z") versus real component of impedance (*Z’)*, which is referredas Nyquist plot.

The complex formalism of the impedance is given by the relation:

$$Z^{*}=\;Z\prime -iZ\prime \prime \;=\;R_{s}\;-\;\frac{1}{j\omega C_{s}}$$

This plot can also be displayed in terms of any of the four possible complex formalisms, the permittivity (ε*), the admittance (Y*), the electric modulus (M*), the impedance (Z*) and dielectric loss (tan*δ*) or dissipation factor. They are related to one another;

$$tan\delta \;=\;\frac{\varepsilon \prime \prime }{\varepsilon \prime }=\frac{M\prime \prime }{M\prime }=\frac{Z\prime }{Z\prime \prime }=\frac{Y\prime }{Y\prime \prime }$$

The Nyquist plots for Gr@ZnO-Nc and ZnO NPs samples were recorded at room temperature and are shown in Fig 8. In the present investigation, flat semicircle was observed for Gr@ZnO-Nc and ZnO NPs between the frequency range 75 Hz–7MHz. The diameter of the semicircle has been reported to correspond to the resistance of the grain [69]. The Nyquist plot of graphene has nearly a vertical line reflecting its high conductivity and rate capability and the plot does not have a semicircle at high frequencies, implying the fast ion diffusion.

This can be attributed to the fact that Z" is inversely proportional to capacitance by the relation;

$$Z\prime \prime =\frac{1}{j\omega C}$$

Moreover, Z' and Z" are given by the relations [69]

$$Z\prime \;=\;\frac{R_{g}}{1\;+\left(\omega_{g}^{2}\;C_{g}^{2}R_{g}^{2}\right)}\;+\;\frac{R_{gb}}{1\;+\left(\omega_{gb}^{2}\;C_{gb}^{2}R_{gb}^{2}\right)}$$

$$Z\prime \prime \;=\;\frac{R_{g}\omega_{g}C_{g}}{1\;+\left(\omega_{g}^{2}\;C_{g}^{2}R_{g}^{2}\right)}\;+\;\frac{R_{gb}\omega_{gb}C_{gb}}{1\;+\left(\omega_{gb}^{2}\;C_{gb}^{2}R_{gb}^{2}\right)}$$

whereR_g_, R_gb_, C_g_, C_gb_ are the resistance and capacitance of grain and grain boundary respectively, while ω_g_ and ω_gb_ are the frequencies at the peaks of the circular arc for the grain and grain boundary respectively. The capacitance and the relaxation times (τ_g_, τ_gb_) can be calculated for the grain and grain boundary by the relations;

$$C_{g}\;=\;\frac{1}{R_{g}\omega_{g}}$$

$$C_{gb}\;=\;\frac{1}{R_{gb}\omega_{gb}}$$

$$\tau_{g}\;=\;R_{g}C_{g}$$

$$\tau_{gb}\;=\;R_{gb}C_{gb}$$

These parameters are obtained by analyzing the impedance data on nonlinear least square (NLLS) fit method and listed in Table 2, which shows the grain boundary resistance R_gb_ decrease while the capacitance C_gb_ increase in the Gr@ZnO-Nc. The presence of a single semicircular arc obtained at higher frequencies corresponds to electrical conduction by the interior of the bulk grain [40]. The data indicated a decrease in the solid state interface layer resistance and the charge transfer resistance on the surface.

**Table 2:** Variation of different impedance parameters as a function of crystalline size.

Sample	Rgb(Ω)	Cgb(nF)	τgb(×10–6 s)
Gr@ZnO-NC	0.46284	181.58	84.0424872
ZnO NPs	1860.9	1.1063	2058.71367

The graphene has been reported to be a competitive candidate for the acceptor material due to its two-dimensional π-conjugation structure,[70] and in the Gr@ZnO-Nc, the excited electrons of ZnO NPs could transfer from the CB to graphene *via* a percolation mechanism[71]. Thus, Gr@ZnO-Nc, graphene served as an acceptor of the generated electrons of ZnO NPs and effectively suppressed the charge recombination, creating more charge carriers to form reactive oxygen species and promote the degradation of MO dye. Overall, both the electron-accepting and transporting properties of graphene in the Gr@ZnO-Nc possibly contribute to the suppression of charge recombination, and thereby a higher rate in the photodegradation is achieved [72].

### 3.7. Gr@ZnO-Nc plausible mechanism for the photocatalytic activity enhancement

The chemical interactions taking place between the charged graphene and the MO dye molecules might have led to the significant adsorption and high photo-degradation. The enhanced photocatalytic degradation of MO dye by Gr@ZnO-Nc could be attributed to the following reasons: (i) the interactions between organic molecules and the aromatic regions of Gr@ZnO-Nc enhance the adsorption on photocatalysts. (ii) the formation of the Zn–O–C chemical bond narrows the band gap of ZnO NPs and efficient photocatalysis and (iii) the transfer of excited electrons from ZnO NPs to graphene suppresses the charge recombination[3].

**Fig 9:**
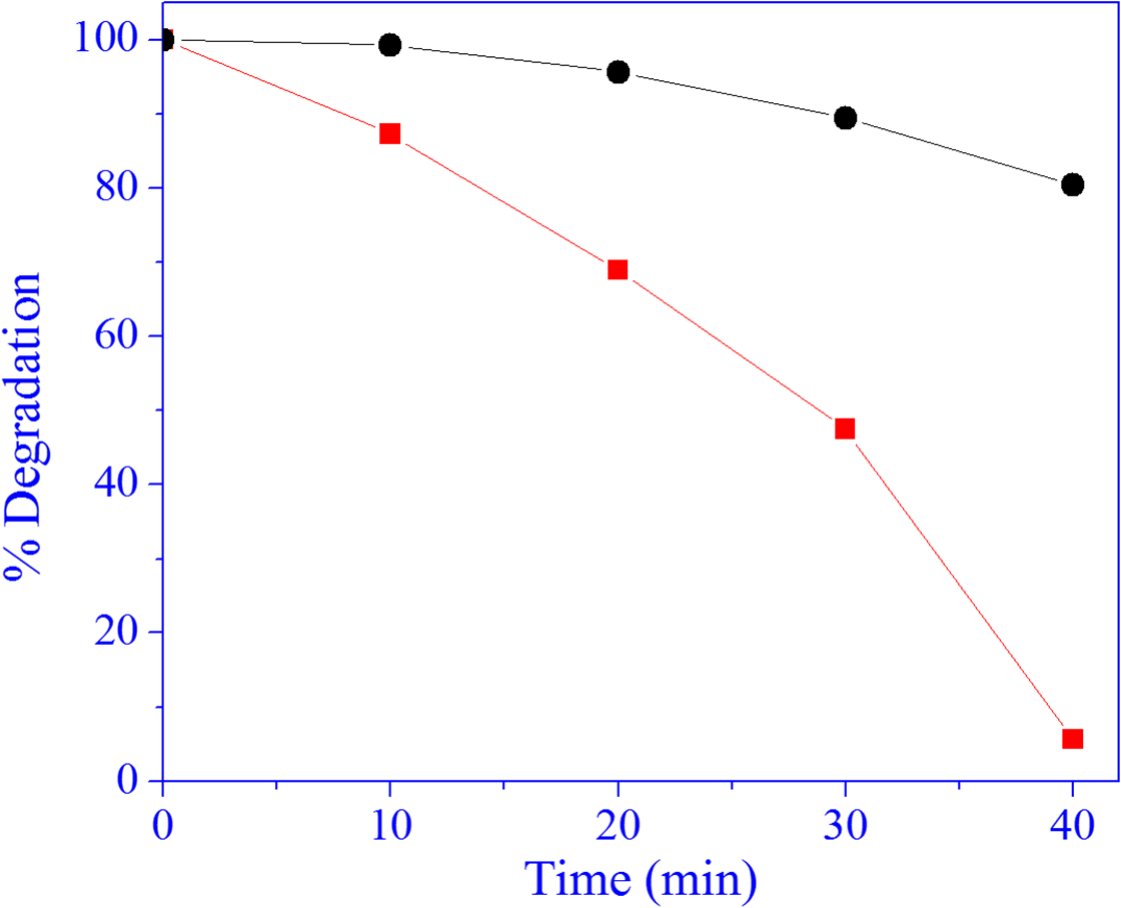
Photo-degradation analysis shows the protective effect of disodium ethylenediaminetetraacetate dehydrate (EDTA-Na_2_; C_10_H_14_N_2_Na_2_O_8_ 2H_2_O) (hole scavenger) (a) and tert-butyl alcohol (C_4_H_10_O) (radical scavenger) (b)on the MO dye in presence of Gr@ZnO-Nc.

The identification of the main active oxidant (reactive oxygen species [73]) in the photocatalytic reaction is of great importance, which helps us to understand the photocatalytic mechanism of Gr@ZnO-Nc. Although, various studies have confirmed that both electrons and holes are the main active species in this system[48, 74–76] while Gr@ZnO-Nc is a photocatalyst capable of producing highly oxidizing ROS [77]. The active oxidants generated in the photocatalytic process of Gr@ZnO-Nc were measured through quenching by disodium ethylenediaminetetraacetate dehydrate (EDTA-Na_2_; C_10_H_14_N_2_Na_2_O_8_· 2H_2_O) (hole scavenger) and *tert*-butyl alcohol (C_4_H_10_O) (radical scavenger) [38, 78]. The data of Gr@ZnO-Nc photocatalyst revealed that the photocatalysis of MO under ultraviolet light exposure was reduced after the addition of EDTA-2Na (Fig 9). This phenomenon suggested that holes are the main active species in this system. The similar photocatalytic mechanism of Gr@ZnO-Nc which is based on the ROS has also been recently reported [76, 77].

**Fig 10:**
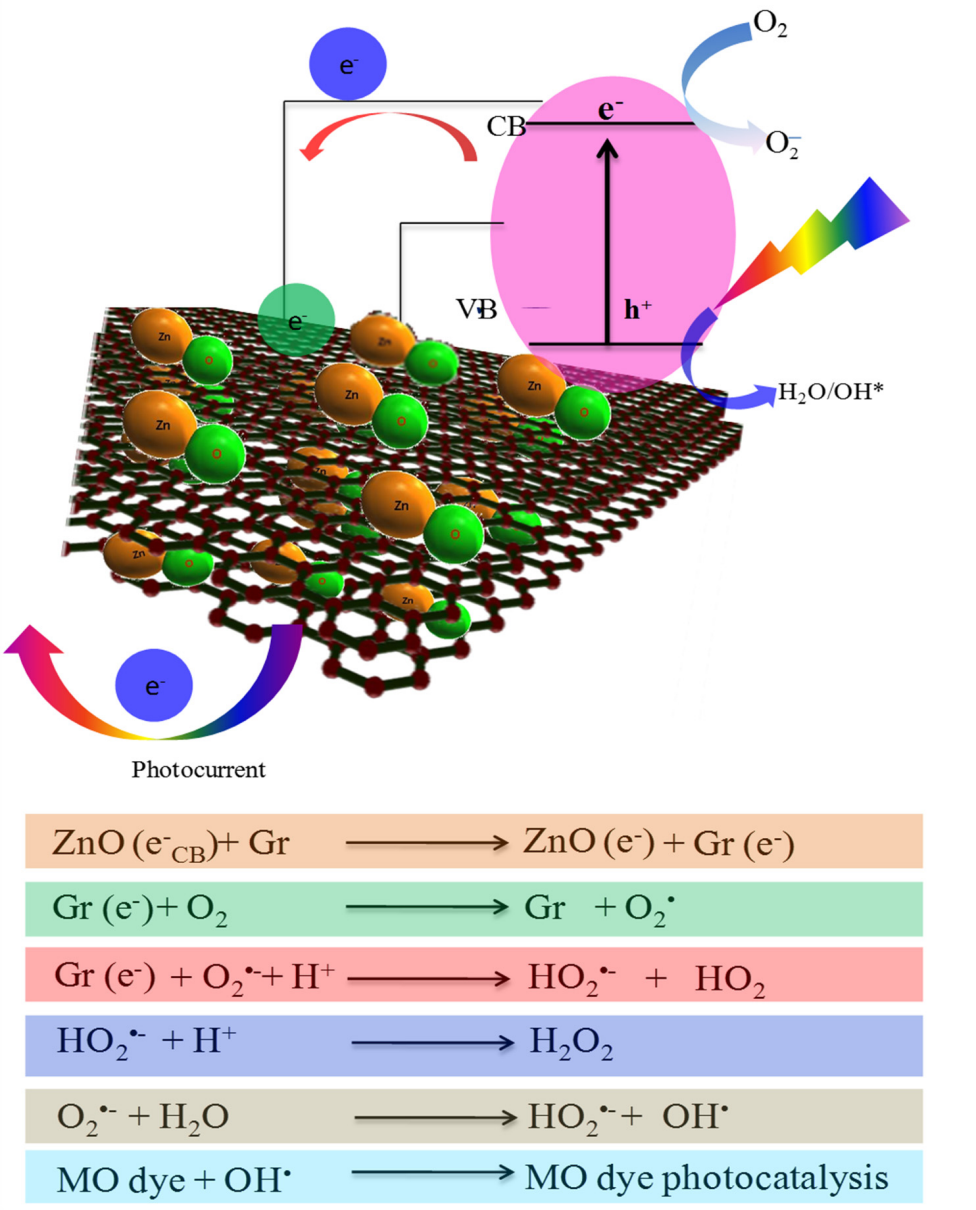
Schematic diagram illustrating the mechanism of photo-degradation MO dye on Gr@ZnO-Nc under exposure to UV light.

The Gr@ZnO NCs serves much higher as a photocatalyst on degrading dyes than the pure ZnO NP. However, bare graphene which are reported to absorb the dyes on its surface by π–π stacking actually interferes and reduce the photocatalytic activity [79]. Thus, the graphene is not directly involved in the photocatalytic activity as a reaction agent. Perhaps, as shown in the in Fig 10, the electronic interaction between ZnO NPs and graphene is one crucial factor responsible for the efficiency of separation of generated charges. The Fig 10 defines that, when a photon with hν energy, which is higher than the E_g_ (i.e. 3.36 eV) value of Gr@ZnO NCs is absorbed it leads to the “promotion” of an electron (e^-^) from the VB of ZnO NPs to the conduction's occurs. This promotes the formation of two charge carriers, namely the e^-^ in the CB, e^-^_CB_, and an empty e^+^ within the VB, which is known as a “hole (h^+^)”. The Gr@ZnO NCs excited states are generated and can start electrochemical chain reaction processes such as redox reactions and molecular transformations of the MO dye. The lower Fermi level of the graphene, the UV-generated e^-^ in ZnO NPs is preferentially transferred to the graphene, leaving the reactive h^+^ behind in the ZnO NPs. This charge separation process effectively reduces the chance of recombination of newly generated e^-^ and h^+^. Therefore, such type of interaction are significantly longer than the life span of h^+^ in Gr@ZnO NCs [80]. This accumulated h^+^ with longer life span can react with the adsorbed water (or surface OH) to form hydroxyl radicals (OH^**.**^), capable of rapid photocatalysis of the MO through the oxidation reaction [81].

## Conclusions

Graphene based nanocomposites have been reported for the great adsorptivity of dyes, extended photoresponding range, and enhanced charge separation and transportation properties simultaneously. Therefore, photocatalytic activity of Gr@ZnO-Nc was studied by the photodegradation of Methylene orange (MO) dye under UV light irradiation. The results revealed that the Gr@ZnO-Nc showed higher photocatalytic activity than ZnO NPs. The synthesized Gr@ZnO-Nc showed more photocatalytic activity than ZnO NPs due to higher density of ZnO NPs in reduced graphene surface due to the generation of large number of photo-induced electron. This work is expected to open a new opportunity in the exploration of Graphene–ZnO NPs nanocomposite and endorse their practical application in addressing various environmental issues.

## Supporting Information

S1 Fig UV-Vis spectrum of sure ZnO NPs. (EPS)

S1 Table Photodegradation efficacy of various Graphene/ZnO nanocomposites materials against dyes. (DOC)
