# Supplementary material for: Kinetic Study on Mutagenic Chemical Degradation through Three Pot Synthesiszed Graphene@ZnO Nanocomposite
Source: PLoS One. 2015 Aug 19;10(8):e0135055. doi: 10.1371/journal.pone.0135055 (PMC4546152; doi:10.1371/journal.pone.0135055)
Supplement: S1 Table — (DOC) [file pone.0135055.s002.doc]

**“Supporting Information”**

| S. No. | Dye | Degradation Time (min) | Refs. |
| --- | --- | --- | --- |
| 1 | Methyl Orange (MO) | 150 |  |
| 2 | Methyl Orange (MO) | 120 |  |
| 3 | Methyl Orange (MO) | 90 |  |
| 4 | Methyl Orange (MO) | 90 |  |
| 5 | Malachite Green (MG) | 90 |  |
| 6 | Methyl Blue (MB) | 60 |  |
| 7 | Methyl Orange (MO) | 40 | Our work |

**Supporting Information Table S1.** Photodegradation efficacy of various Graphene/ZnO nanocomposites materials against dyes.
